# Quality Assessment of the Protein Ingredients Recovered by Ultrasound-Assisted Extraction from the Press Cakes of Coconut and Almond Beverage Preparation

**DOI:** 10.3390/foods11223693

**Published:** 2022-11-18

**Authors:** Gilda Aiello, Ruoxian Xu, Raffaele Pugliese, Martina Bartolomei, Jianqiang Li, Carlotta Bollati, Lukas Rueller, Josef Robert, Anna Arnoldi, Carmen Lammi

**Affiliations:** 1 Department of Human Science and Quality of Life Promotion, Telematic University San Raffaele, 00166 Rome, Italy; 2 Department of Pharmaceutical Sciences, University of Milan, 20133 Milan, Italy; 3 NeMO Lab, ASST Grande Ospedale Metropolitano Niguarda, 20162 Milan, Italy; 4 Fraunhofer Institute for Environmental, Safety and Energy Technology UMSICHT, 46047 Oberhausen, Germany

**Keywords:** food by-products, food waste, plant protein, press cakes, ultrasound-assistant extraction, vegetal drinks

## Abstract

The manufacture of vegetal beverages has the drawback of producing large amounts of press cakes that are generally used as feed components. This work had the objective of valorizing the press cakes deriving from almond and coconut drinks production by using ultrasound-assisted extraction (UAE) to obtain protein ingredients for human use. Starting from coconut and almond press cakes, whose initial protein contents were 19.7% and 18.6%, respectively, the UAE treatment allowed liquid fractions to be obtained that were then freeze-dried: the extraction yields were 24.4 g dry extract/100 g press cake in case of coconut and 49.3 g dry extract/100 g press cake in case of almond. The protein contents of these dried materials were 30.10% and 22.88%, respectively. The quality of the extracted protein ingredients was assessed in term of phytic acid content, protein profile, techno-functional features, and antioxidant properties. The sonication had also a favorable effect on digestibility.

## 1. Introduction

The interest for a plant-based diet is continuously increasing owing to the consumer demand caused by health concerns and vegetarianism trends as well as by sustainability reasons [1,2]. In particular, the market of vegetal beverages is becoming larger and larger, because these products are attractive alternatives to cow’s milk for vegans, vegetarians, and flexitarians as well as for people with lactose intolerance or cardiovascular disease. Their manufacturing procedure involves pressing the starting material into the ground in the presence of water; the liquid phase is the final beverage, whereas the solid phase constitutes a residual press cake that still contains a certain amount of protein. These by-products are mainly used as feed components, although their use in human nutrition would be much more desirable and profitable. Coconut and almond are widely used for producing vegetal drinks and their press cakes are among the by-products that would require a better valorization [3]. Efforts are therefore needed to identify new strategies for an added-value use of these materials in order to achieve the final goal of a more sustainable process for their production. In this context, it is useful to underline that literature indicates that almonds contain 20–22% (w/dry w) protein providing all essential amino acids (mainly leucine, valine, and phenylalanine) and a very high intake of arginine [4], whereas coconut contains 5–6% (w/dry w) protein with a relatively favorable amino acid profile, containing all essential amino acids (mainly leucine, lysine, and valine) [5]. Since part of these proteins remains in the press cake, a main priority is certainly the development of new methods for the recovery of protein-rich ingredients from these by-products.

Among the numerous strategies used to improve the extraction yields of proteins and other bioactive components from food by-products, ultrasound-assisted extraction (UAE) appears to offer a useful solution because this technology can accelerate mass transfer and enhance the extraction kinetics [6]. The mechanical ultrasound waves cause pressure changes within the liquid medium and hence lead to acoustic cavitation. Microbubbles are thus generated, grow during sonication, and implode as soon as they reach a critical diameter. The instant implosion causes numerous physical effects, such as high shear forces and turbulent conditions within the liquid. As a consequence, cell structures are disintegrated, and extraction processes are enhanced. The UAE is cheaper and easier to operate than other novel extraction techniques, such as pressurized liquid extraction (PLE) and microwave-assisted extraction (MAE) [7]. Moreover, it permits the extraction of a wide variety of natural compounds from food matrices, including proteins, due to the cavitation effect that enhances mass transport by disrupting the plant cell walls [8] and its application is not restricted by the solvent or type of matrix used. The ultrasonic power is one of the key factors leading to an efficient extraction [9].

As for the consequence on protein integrity, some studies have investigated the structural changes after ultrasound treatment. In general, the increased intra-molecular mobility causes changes in the free sulfhydryl groups, particle size, surface hydrophobicity, and secondary structure organization [10], although minimal structural variations are reported for whey proteins after ultrasonication [11]. It is, however, important to underline that several literature reports are focused on long-term or continuous sonication processes, whereas the effects of short-time or intermittent sonication are so far scarce. In this context, this work had the objective of evaluating the effects of UAE on the quality of the proteins extracted from almond and coconut press cakes. To work in an energy-efficient manner, the ultrasonication was performed at high intensity and for a short time, in operational conditions (time, frequency, and temperature) that had been optimized in preceding experimentations performed on soy press cake (soy okara) [12]. The protein integrity was evaluated using different analytical methods, also including high-performance liquid chromatography coupled with tandem mass spectrometry using either an untargeted or a targeted method to investigate the variations in the primary structures of extracted proteins. Finally, the techno-functional properties were evaluated as well as the antioxidant properties.

## 2. Materials and Methods

### 2.1. Chemicals

All reagents were of analytical grade. Acetonitrile (ACN), tris(hydroxymethyl)aminomethane (Tris-HCl), 2-iodoacetamide (IAM), 1,4-dithiothreitol (DTT), trypsin from bovine pancreas (T1426, lyophilized powder, ≥10,000 units/mg protein), diphenyl-1-picrylhydrazyl (DPPH), 2,2-azino-bis-(3-ethylbenzothiazoline-6-sulfonic acid (ABTS), 2,4,6-Tris(2-pyridyl)-s-triazine (TPTZ), azo 2,2′-azobis(2-methylpropionamidine) dihydrochloride (AAPH), Trolox, fluorescein were from Sigma-Aldrich (St. Louis, MO, USA). Mini-Protean apparatus, precision plus protein standards, Bradford reagent, and Coomassie Blue G-250 were purchased from Bio-Rad (Hercules, CA, USA).

### 2.2. Press Cake Samples and UAE Treatment

The almond and coconut press cakes were supplied by a German company as frozen materials in 3 kg units directly from the production process. The protein content of the coconut press cake was 19.67% and that of the almond press cake was 18.56%. The ultrasound-assisted protein extraction experiments were performed at Fraunhofer UMSICHT, Germany, using an ultrasonic continuous flow system (TC 10, BSONIC GmbH, Halver, Germany). The reactor volume was 4.6 L and the probe tip had a diameter of 41.75 mm. The defined ratio of 1:2.5 of press cake and tap water was heated up to a temperature of 60 °C under continuous stirring. Then, the mixture was pumped into the ultrasound reactor at a flow rate of 140 L/h (membrane pump); the ultrasound parameters were fixed as follows: power input 4.5 kW, frequency 18 kHz, oscillation amplitude 45–60 µm, and the residence time 2 min. Hence, the total energy input for each sample was 117 kJ/L. The regulation of power input and recording of process parameters were performed via PC. The raw and ultrasonicated press cakes were analyzed by scanning microscopy to investigate their morphologies. Afterwards, the processed samples were separated mechanically via a sieving press (150 µm) receiving a solid, which was discarded, and a liquid fraction that were separately freeze-dried (Alpha 2–4 LSC plus, Christ). All liquid fractions were freeze dried according to a specific program for protein containing liquids. Vacuum pressure was applied at 0.1 mbar during the entire program. The freeze-drying duration was 60 h in total. The temperature was set at −20 °C for 10 h, −15 °C for 10 h, −10 °C for 5 h, 0 °C for 10 h, 10 °C for 10 h, and 20 °C for 15 h. The extraction yields were 24.4 g dry extract/100 g press cake in case of coconut and 49.3 g dry extract/100 g press cake in case of almond. The protein contents of these dried materials were 30.10% and 22.88%, respectively. These dried materials were then submitted to a series of analyses.

### 2.3. Scanning Electron Microscopy (SEM) of Raw and Treated Press Cakes

Scanning electron microscopy (SEM) was performed with a Vega-3 microscope (TESCAN GmbH, Dortmund, Germany), at 20 kV acceleration voltage under high vacuum. To guarantee a high resolution, the images were obtained with a secondary-electron detector (SE). Freeze-dried samples (raw and treated press cakes) were fixed on a special two-sided adhesive tape and coated by a 10 nm gold surface to prevent electrostatic charging.

### 2.4. Phytic Acid Determination

Phytic acid was analyzed in the freeze-dried liquid fractions and raw press cakes, following a modified colorimetric method [13]. The concentration range of 0–100 μg/mL of aqueous phytic acid was used as the standard for quantification. Aliquots of samples and standards (100 μL) were diluted 25 times with 2.4 mL of H_2_O; 600 μL of the diluted samples and standards were combined with 200 μL of modified Wade reagent (0.03% of FeCl_3_ 6H_2_O and 0.3% of sulfosalicylic acid), and the absorbance was measured at 500 nm by using a SmartSpec™ Plus Spectrophotometer.

### 2.5. Protein Profiling by SDS-PAGE

To compare the effects of UAE on the protein profiles, either the raw press cakes and the freeze-dried liquid fractions were extracted with 0.1 M Tris-HCl buffer (pH 8.2, 0.5 M NaCl) by using a 1:15 (*w/v*) ratio, stirring for 2 h at 4 °C. The mixtures were then centrifuged at 8000× *g* for 15 min, the supernatants were collected and the protein concentrations were determined by the colorimetric Bradford test, carrying out the analyses at a wavelength of 595 nm and employing a standard curve based on BSA. These samples were analyzed by SDS-PAGE, using a 12% of polyacrylamide gel and a Tris-glycine buffer (pH 8.3, 0.1% SDS), and then submitted to the following analyses as described beneath.

### 2.6. Discovery Proteomics by HPLC-Chip ESI-MS/MS

The protein profile was investigated in detail by untargeted MS analysis after in-solution tryptic digestion. An equivalent volume of protein extract and 50 mM of NH_4_HCO_3_ was mixed. The reduction and subsequently alkylation was carried out according to a previously published study [14]. Then, 4 mg/mL of trypsin solution were added to the processed samples with a ratio of 1:50 (*w/w*, E/S) and the reaction mixture was incubated for 16 h at 37 °C. Formic acid (FA, 0.1%) was used to block the reaction by adjusting the pH to 3–4. Digested samples were purified using Sep-Pak C18 cartridges (Thermo Fisher Scientific, Life Technology, Milan, Italy), and analyzed by LC-MS according to Aiello et al. [12]. The Spectrum Mill Proteomics Workbench (Rev B.04.00, Agilent Technologies, Santa Clara, CA, USA) was used for the automated peptide identification from tandem mass spectra, consulting the *Cocos nucifera* (599) and *Prunus dulcis* (41767) databases. Trypsin was selected as the cutting enzyme with two allowed missed cleavages and carbamidomethylation was chosen as a fixed modification. The mass tolerance was set at 1.0 Da and 0.8 Da for MS1 and MS2, respectively. An auto-validation strategy for both peptide and protein polishing mode was performed using FDR cut-off ≤1.2%.

### 2.7. Quantitative Evaluation of the UAE Effects by MRM Analysis

A more precise evaluation of the effects of the UAE process on the protein quality was carried out by a quantitative MRM assay. Specifically, unique peptides belonging to some main coconut and almond proteins (a legumin and a vicilin for coconut and a legumin for almond) were selected using the Skyline software (version 20.1, 64-bit, Seattle, WA, USA) with the setting described in a published paper [15]. In silico digestion was achieved by using the following parameters: enzyme, trypsin; peptide length, 7–25 amino acids long missed cleavages, none. Peptides containing cysteine and methionine were omitted from the analysis due to their possible modifications. For MRM analysis, the following “transition settings” (settings > transition settings) parameters were used: precursor charges, 2; fragment ion charges, 1; ion type, y and b; product ions, 3.

### 2.8. Free Sulfhydryl Content

The free sulfhydryl content was determined by a literature method [16] with appropriate adjustments. Protein extracts (20 μL) were dissolved in 180 μL Tris-glycine buffer solution (0.086 mol/L Tris, 0.09 mol/L glycine, 4 mmol/L ethylenediaminetetraacetic acid, 8 mol/L urea, pH 8.0). Then, 5,5′-dithiobis(2-nitrobenzoic acid) (DTNB, 5 μL) was added to the mixed solution, the mixture was reacted for 15 min at room temperature and the absorbance was measured at a wavelength of 412 nm. Glutathione (0–0.16 μmol/mL) was used as a standard to prepare the calibration curve. The mixture solution without DTNB was used as a control. The free sulfhydryl contents (-SH) were calculated as follows:

Free SH (μmol/g) = [(A − b)/(a × C)] × 10 × 1000

where a and b terms are from the equation of the standard curve y = ax + b; A is the absorbance of the samples; C is the protein concentration (mg/mL); and 10 is the dilution factor.

### 2.9. Circular Dichroism

CD spectra were recorded in a continuous scanning mode (190–250 nm) at 25 °C using a Jasco J-810 (Jasco Corp., Tokyo, Japan) spectropolarimeter. The experimental conditions used were previously described [12]. The estimation of the peptide secondary structure was achieved by using the DichroWeb site (http://dichroweb.cryst.bbk.ac.uk/html/home.shtml accessed on 1 September 2021) [17].

### 2.10. Intrinsic Fluorescence

The intrinsic fluorescence spectra were obtained using a fluorescence spectrophotometer (Synergy H1, Biotek). The samples were diluted in phosphate-buffered saline (PBS, 10 mM, pH 7.0) in order to reach the equal concentration of 0.05 mg/mL and were transferred in Greiner UV-Star^®^ 96 well plates with flat bottom clear cyclic-olefin copolymer (COC) wells. The excitation wavelength was set as 280 nm and the excitation and emission slit widths were set as 5 nm. The emission wavelength range was set up from 300 nm to 450 nm and the scanning speed was 10 nm/s.

### 2.11. Micro near InfraRed (NIR) Spectroscopy

NIR spectra were recorded on a MicroNIR device (Viavi Solutions, JDSU Corporation, Milpitas, CA, USA) operating in the spectral region 900–1700 nm. This is an ultra-compact device consisting of a linear variable filter (LVF), as dispersing element, directly connected to a 128-pixel linear indium gallium arsenide (InGaAs) array detector and two tungsten light bulbs as radiation source. Collection of spectra was performed with a nominal spectral resolution of 6.25 nm, as the most performing condition, using a special tool designed to obtain the optimal focal point in order to improve the chip sensitivity. The spectra ion was used as the NIR standard reference with a 99% diffuse reflectance. All collected spectra were recorded with an integration time of 10 ms, resulting in a total measurement time of 2.5 s per sample.

### 2.12. Determination of Protein Solubility, Water Binding Capacity (WBC) and Oil Binding Capacity (OBC)

The protein solubility (PS) was determined according to a method previously described [18] with small modifications. Each sample (0.1 g) was dispersed into 2 mL of 0.1 M phosphate buffer solutions (at pH values from 2.0 to 10.0) and stirred for 20 min at RT. After the pH adjustment, the samples were stirred 30 min at RT and then centrifuged at 14,000 rpm for 30 min. The protein concentration in the liquid phase was determined according to the Bradford assay using BSA as a standard. The PS was expressed as percentage ratio of supernatant protein content to the total protein content. All determinations were conducted in triplicate. The WBC was determined according to a method previously described [12]. In details, 50 mg of samples was dispersed in 500 μL distilled H_2_O_2_ and vortexed for 1 min. The mixture was incubated at room temperature for 30 min and then centrifuged at 7000× *g* for 25 min at RT. The resulting supernatant was carefully decanted, and the tube containing the precipitation weighed. The OBC was determined according to a literature method [18]. In details, 50 mg of samples was dispersed in 500 μL sunflower oil and vortexed for 1 min. The mixture was incubated at room temperature for 30 min and then centrifuged at 5000× *g* for 20 min at RT. The resulting supernatant was carefully decanted, and the tube containing the precipitate was weighed.

### 2.13. Foaming Properties

The foaming properties were determined in triplicate using a literature method [19] with slight modifications. Solutions containing 1% protein were prepared in distilled water. Aliquots of 50 mL (V1) were blended for 5 min using a magnetic stirrer (VELP Scientifica Srl, Usmate Velate, Italy) at the highest speed, poured into 250 mL graduated cylinders, and the volume of foam (V2) was immediately recorded at 0, 5, 30, and 60 min. The foaming was calculated using the following equation: Foaming = (V2−V1) × 100/V1. The foaming capacity was determined at 0 min and the foam stability (FS) after 5, 30, and 60 min.

### 2.14. In Vitro Protein Digestibility (IVPD)

With a two-stages digestion procedure, two enzymes (pepsin and pancreatin) were applied to simulate the in vivo protein digestion in the gastrointestinal tract [20]. During the first digestion stage, the pH of the protein extract (500 μL) was adjusted to 2–3 using 1 M HCl and then 5 μL of pepsin solution (10 mg pepsin/mL in 0.01 M HCl) was added. After 30-min incubation at 37 °C, 5 μL of 1.0 M NaOH solution was added to stop the hydrolysis and adjust the pH to 7.8. Then, 15 μL of pancreatin solution (10 mg/mL in H_2_O, pH 7.0) was added to continue the digestion incubating the mixture for 1 h at 40 °C. Finally, 5 μL of Na_2_CO_3_ solution (150 mM) was added to stop the reaction. During this procedure, 20 μL of the initial protein extract solution, pepsin digested protein solution, and pancreatin digested protein solution were collected. These samples were centrifuged at 5000× *g* for 10 min, and the supernatants were collected for peptide and protein measurement using Bradford assay. The in vitro protein digestibility was calculated using the following equation:

$$IVPD\;\left(\%\right)=\frac{P0-P1}{P0}\times 100$$

where *P*_0_ is initial protein content, *P*_1_ is the final undigested protein content.

### 2.15. Evaluation of the Antioxidant Properties

Three different assays were applied. The 1,1-diphenyl-2-picrylhydrazyl radical (DPPH) assay was performed by a standard method with slight modifications [21]. The TEAC assay, based on the reduction of the 2,2-azino-bis-(3-ethylbenzothiazoline-6-sulfonic acid (ABTS) radical was performed as previously described [21] as well as the ferric reducing ability (FRAP) assay [21].

### 2.16. Statistical Analysis

All experiments were performed in triplicate and the results were presented as the mean  ±  standard deviation. The collected data were subjected to analysis of variance (ANOVA) and Duncan’s multiple range test was used to analyze differences between treatments (*p*  <  0.0001).

## 3. Results

### 3.1. Ultrasound-Assisted Extraction

As already indicated in the introduction, the almond and coconut press cakes were treated with ultrasound in conditions (time, frequency, and temperature) that had been optimized in a preceding investigation with the scope of improving the protein extraction yields while preserving the protein integrity as much as possible [12]. The UAE was thus performed at high intensity (power input 4.5 kW, frequency 18 kHz, oscillation amplitude 45–60 µm), at 60 °C and for a short residence time (2 min). The chemical compositions of the wet almond and coconut press cakes before the application of UAE have been previously reported elsewhere [22], as well as the composition of the wet UAE liquid phases [23]. Specifically, the % dry matter of the almond liquid fraction was 0.02 ± 0.0004%, the % ash content was 0.009 ± 0.0003%, the soluble protein content was 0.038 ± 0.001 mg/mL, and the reducing sugars content was 0.002 ± 0.001 mg/mL. The % dry matter of the coconut liquid fraction was, instead, 0.03 ± 0.0002%, the % ash content was 0.015 ± 0.009%, the soluble protein content 0.028 ± 0.0007 mg/mL and the reducing sugars content was 0.004 ± 0.0007 mg/mL. The protein content and the dry matter were also determined on both almond and coconut dried press cakes and on freeze-dried liquid fractions obtained after the UAE. The almond and coconut press cake dry matter contents were 23.8% and 24.3%, respectively. Starting from coconut and almond press cakes, whose initial protein contents were 19.7% and 18.6%, respectively, the UAE treatment, followed by a simple sieve filtration, allowed liquid fractions to be obtained that were then freeze-dried in a 24.4% yield in case of coconut and a 49.3% yield in case of almond. The protein contents of these materials were 30.10% and 22.88%, respectively. The increase in the protein content of both materials underlines the efficiency of the UAE for recovering protein rich materials from food by-products. This was particularly true with the coconut.

### 3.2. Effects on the Morphology of the Coconut and Almond Press Cakes

**Figure 1:**
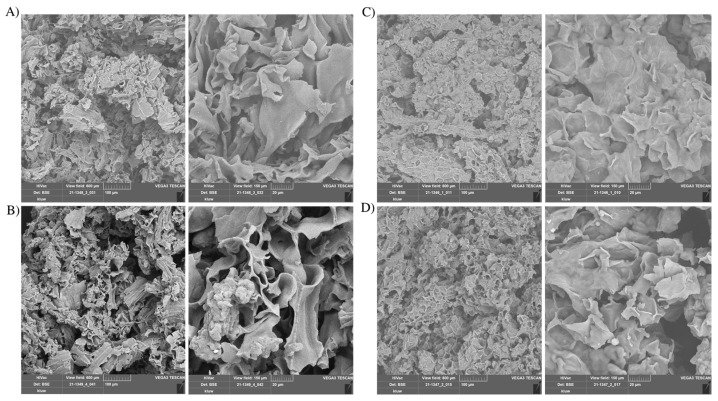
Comparison by the SEM of raw and ultrasound treated press cakes at two different magnifications (left 300× and right 1500×). (**A**) Raw coconut press cake; (**B**) ultrasound treated coconut press cake; (**C**) raw almond press cake; (**D**) ultrasound treated almond press cake.

Scanning electron microscope analysis was used to compare the morphology of the press cakes before and after the ultrasonication process (Figure 1). Whereas the structures of the raw coconut (Figure 1A) and almond (Figure 1C) press cakes were regular and compact, those of the sonicated materials showed a slight degree of disintegration, mostly visible in the coconut sample (Figure 1B), while the almond sample appeared to be less changed (Figure 1D). This may explain the higher protein content of the coconut liquid phase.

### 3.3. Phytic Acid Content

Phytic acid (PA) is a well-known undesirable antinutritional factor. The literature indicates that several processing methods, such as soaking, malting, fermenting, and heat treatments, may be useful for reducing its content [24], although none of these techniques results in the complete removal of PA. Since some evidence also supports a favorable effect induced by ultrasound treatments [25], it was decided to measure the residual PA contents of the protein-rich liquid fractions. Indeed, our findings demonstrate that the process induced a 44.4 ± 1.0% reduction (**** *p* < 0.0001) of the PA content of the almond sample (from 2.16 ± 0.04 mg/g of protein in raw press cake to 1.21 ± 0.01 mg/g of protein in the UAE liquid fraction), and a 12 ± 0.5% reduction in its contents in the coconut (from 1.05 ± 0.01 mg/g of protein in the raw press cake to 0.92 ± 0.03 mg/g of protein in the UAE liquid fraction). The smaller reduction observed with coconut may depend on the lower content of this antinutrient in the raw press cake. The PA reduction may be explained by a disruption of the phosphate bonds with the liberation of inositol induced by the cavitation as well by the dissolution of water-soluble PA salts and the activation of endogenous phytases during soaking and ultrasonication [26].

### 3.4. Protein Profile

**Figure 2:**
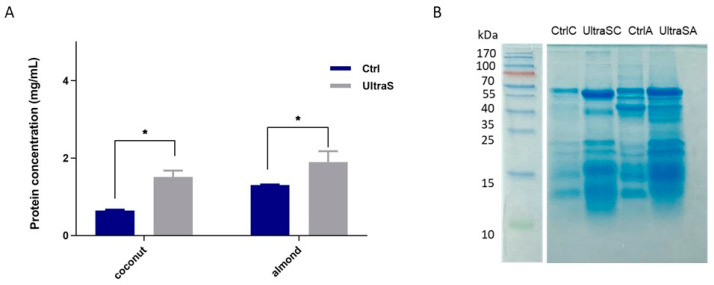
(**A**) Comparison of the “soluble protein” contents of coconut and almond samples: raw press cakes (Ctrl) and UAE treated freeze-dried liquid fractions (UltraS), * significantly different (*p* < 0.05) versus the raw press cake. (**B**) Reduced SDS-PAGE protein profile of raw press cakes (CtrlC and CtrlA) and the treated liquid fractions (UltraSC, UltraSA); M, pre-stained molecular marker. Each sample (10 μL) was added to 10 μL of loading buffer, loading 20 μL for each well.

The following experiments were dedicated to compare the protein quality in the raw press cakes and in the freeze-dried liquid fractions. In order to do so, the proteins were extracted from both materials with a standard laboratory procedure [0.1 M of Tris-HCl buffer, pH 8.2, 0.5 M NaCl, using an 1:15 (*w/v*) ratio] and then submitted to a series of analyses. In the Figure 2, the proteins extracted from the coconut and almond press cakes are indicated as CtrlC and CtrlA, whereas those extracted from the coconut and almond freeze-dried liquid fractions are indicated as UltraSC and UltraSA, respectively. The protein concentrations of these materials provide a rough comparison of the amount of “soluble proteins” in the raw press cakes and the liquid samples. Figure 2A shows that these parameters were increased by the UAE in both cases. With coconut, a 132% increase was observed (from 0.65 ± 0.03 mg/mL in the raw sample to 1.51 ± 0.16 mg/mL in the treated sample), whereas with almond, a smaller 53% increase was observed (from 1.30 ± 0.02 mg/mL in the raw sample to 1.89 ± 0.27 mg/mL in the treated sample). These results agree with the higher protein contents of the liquid phases versus the raw press cakes measured by the Kjeldahl method.

These findings are comparable to those reported on other food materials, such as soy, whey, and egg white treated by UAE [27]. In particular, different authors have observed that an increase in sonication time and intensity (W/cm^2^) or power (W) up to an optimal value may improve the solubility of the 7S fraction of a soy protein isolate (SPI). The best conditions were 400 W/cm^2^ for 40 min [28]; 131–138 W/cm^2^ for 30 min (Hu et al., 2013c); or 200 W for 30 min and 251.16 W for 60 min [29].

However, the UAE may produce changes in the primary structures due to the cleavage of peptide bonds or, on the contrary, due to aggregation. For elucidating this aspect, the protein profile was investigated by SDS-PAGE as shown in Figure 2B. In detail, the absence of bands at the top of the separation gel and loading port confirms that no protein aggregates were formed upon the ultrasonication. Moreover, no clear signs of the cleavage of the protein primary structure were observed in the ultrasonicated samples versus the raw materials. Instead, more intense bands along the entire range of molecular weights were observed in the lanes of the ultrasonicated samples: this evidence is stronger in the coconut sample in agreement with the sharp rise in the protein content after ultrasound treatment. This suggests that the UAE disrupts the food matrix thus permitting the release of otherwise less accessible proteins. Our results agree with a published paper where the soy protein yields were improved by 20, 23, 30, and 46%, respectively, versus the control, after sonication times of 15, 30, 60, and 120 sec at a frequency of 20 kHz [30]. However, the different treatment conditions used in that paper suggest that the total energy/energy input was much lower than in our case. A recent paper [31] has shown no difference in the primary structure of the protein profile of untreated and ultrasound-treated egg white, with a treatment conducted at 55 kHz, 45.33 Wcm^−2^ for 12 min.

### 3.5. Proteomic Analysis

The proteins of the UAE liquid fractions and raw press cakes were then subjected to discovery proteomics by LC-MS analysis to fully characterize the proteome compositions. The results of the data-dependent acquisition (DDA) analysis are reported in Table S1. The most abundant storage proteins, i.e., vicilin and legumin, were easily detected either in the raw almond press cakes or the ultrasonicated liquid sample, whereas in both coconut samples, the most abundant protein was cocosin, the main component of legumin.

In addition to storage proteins, some membrane proteins such as non-specific lipid-transfer protein and putative lipid transfer protein (fragment) were detected in CtrlA, whereas proteins involved in the response to water stimuli, i.e., dehydrin xero 1 (fragment) and abscisic acid response protein, were identified in UltraSA. Besides storage proteins, i.e., 11S globulin and cocosin, in UltraSC and CtrlC cytoplasmatic proteins such as ribosomal protein L2, non-specific serine/threonine protein kinase, acyl-[acyl-carrier-protein] hydrolase and nuclear proteins, such as DNA-directed RNA polymerase subunit, were detected. These results indicate the presence of a subset of proteins involved in metabolic processes, such as binding with regulatory enzymes.

### 3.6. Label-Free Multiple Reaction Monitoring for the Quantification of Main Storage Proteins

In order to obtain further insight into the changes induced by the UAE treatment, it was decided to develop a method for the quantification of the most abundant storage proteins detected by DDA MS analysis. The ideal choice of peptides and their transitions is critical for the sensitivity and selectivity of an MRM experiment. The proteotypic peptides and their transitions were forecast by the Skyline software ver. 20.1, according to the following criteria: (i) peptides containing 7 to 25 residues, (ii) peptides with double or triple charged *m/z* values within the mass range of the instrument, (iii) peptides without modification sites or amino acids susceptible to variable changing during the sample processing. Three peptides for each protein and the relative transitions calculated with the Skyline software are listed in Table 1. The generated transition list was then exported, and the MRM method was set up on the IT instrument. Two transitions per peptide were monitored simultaneously for quantitative and qualitative analysis of the target proteins in coconut and almond samples.

Nominal peak areas in the extracted ion chromatogram (XIC) were adopted for a relative quantification of each peptide. No calibration curve was used and the ratio between the areas of each peptide in the UltrS and Ctrl samples was adopted for quantification. The average ratios of each peptide per proteins (Table 1) showed that the intensity of the main proteins in coconut and almond increased after the ultrasound treatment. These results will be discussed here using the average of the ratios of the three peptides monitored for each protein. In coconut, the legumin was 3.7-fold more abundant and the vicilin 1.8-fold was more abundant in the UAE treated sample than in the raw press cake, whereas in almond, the 11S globulin isoform 2 was 4.1-fold more abundant in the UAE treated sample than in the raw press cake. These outcomes confirm more accurately what had been highlighted by SDS-PAGE.

### 3.7. Secondary and Tertiary Structure of Coconut and Almond Proteins

Although the protein primary structures did not change much, low-frequency and high-intensity ultrasonication may induce changes in the secondary and tertiary protein structures due to the cavitation-induced shear leading to protein aggregation and cross-linking due to oxidation.

**Figure 3:**
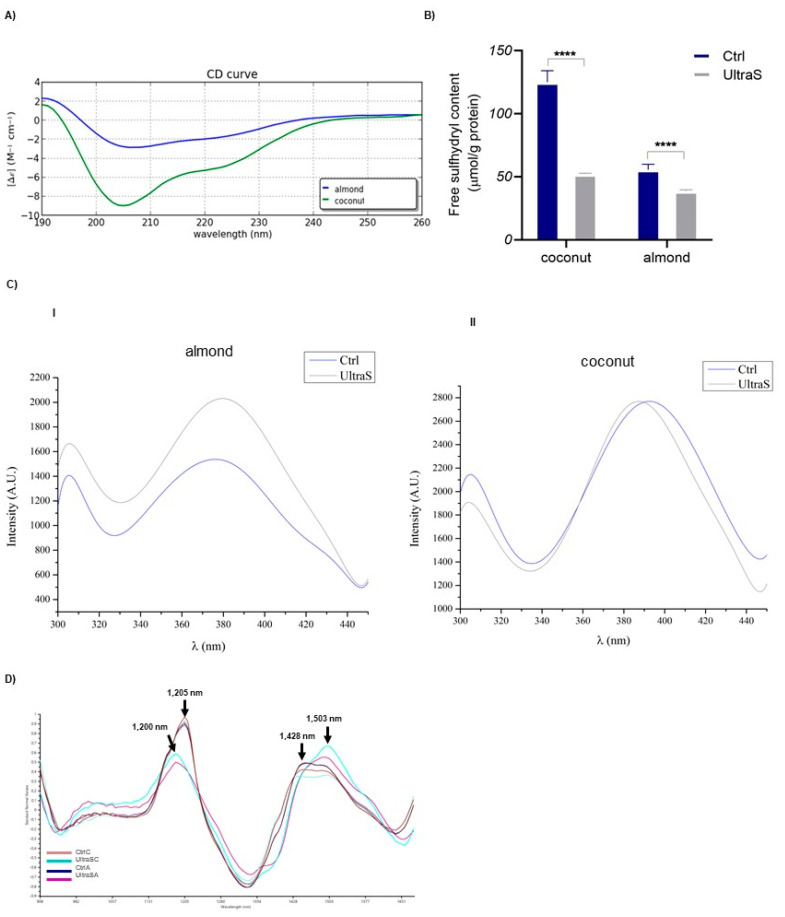
(**A**) CD spectra of almond and coconut UltraS; (**B**) free SH content in UltraSC and UltraSA, CtrlC and CtrlA, (****) *p* < 0.0001.; (**C**) intrinsic fluorescence signal detection of almond (**I**), and coconut (**II**); (**D**) micro-NIR spectra expressed as standard normal variate (SNV).

Hence, CD spectroscopy was used to quantify the changes of the protein secondary structure induced by the ultrasound processing. The results are summarized in Table 2 and Figure 3A. Globally, it may be affirmed that these kinds of proteins have a low content (around of 2–3%) of α-helix, about 25–30% of β-sheet substructures, 20–25% turns, and over 40% of random coils. After ultrasound processing, the CD spectra indicated a small decrease of α-helix percentage (larger in almond proteins), an evident decrease of the β-sheet in both samples, an increment of turn only in the case of almond, and a general increase in random coils. Remembering that these CD spectra reflect the situation of very complex protein mixtures, it seems possible to affirm that the UAE produced only a moderate de-structuring of the protein conformation. This is very important, since the α-helix and β-sheets are the major substructures responsible for the maintenance of the tertiary protein structure, which affects important techno-functional properties, such as the solubility, swelling ability, and viscosity.

The changes in the protein secondary structures might be due to shear forces, shock waves, and turbulence caused by ultrasonication. In fact, the cavitation disrupts the interactions between the local sequences of amino acids and between different parts of the protein molecule, resulting in changes in their secondary structures [32]. Furthermore, ultrasound application may lead to selective disruption of hydrogen bonds resulting in secondary structure changes depending on the local protein conditions and amino acid sequence [33]. However, it is well documented that the specific conditions of ultrasonication, i.e., the time and the power, may affect the secondary structure rearrangement in terms of changing the α-helix and β-turn content, indicating that over-processing with ultrasonic waves might increase the extensions of certain types of hydrogen bonds as verified for the β-lactoglobulin [34].

The measurements of the content of free SH groups located on the surface of coconut and almond proteins as well as the detection of their intrinsic fluorescence spectra were used to provide further insight into the ability of sonication to cause changes in the protein tertiary structure. Evidence in the literature indicates that ultrasonication may change the number of free SH groups, by exposing buried SH groups initially located inside the protein [27]. Our findings, instead, demonstrate a significant reduction in the number of free SH group either in the coconut or almond ultrasonicated proteins (Figure 3B). This is particularly evident in coconut, where the free SH content of 125.15 ± 9.0 μmol/g of the raw press cake was reduced to 52.25 ± 0.58 μmol/g in the proteins submitted to the UAE.

These findings clearly indicate that the UAE significantly impacts on the structure of the protein with a reduction in the exposition of the cysteine residues, probably due to the formation of intermolecular disulfide bonds S-S, which modulate the folding of the extracted proteins. This trend has been also observed on soy okara proteins [12] after a UAE treatment performed in the same conditions. A similar decrease has also been observed in 7S and 11S soy proteins treated at 400 W for 5, 20, and 40 min [35]. This phenomenon may possibly depend on the generation of radical species during the sonication process, where water molecules may dissociate to generate substantial amounts of hydroxyl radicals that can oxidize the free SH groups to S-S bonds [36]. However, the divergences in the content of SH groups and S-S bonds may depend also on the sonication parameters and time as well as on the presence of impurities.

Further information on the modification of the tertiary structure was obtained applying intrinsic fluorescence spectroscopy that allows to detect the fluorescence due to tryptophan (Trp) [37]. The fluorescence spectra were recorded in the range 300–460 nm. The spectra of the almond samples (Figure 3C-I) showed a main peak at 380 nm, whose intensity increases by 27.2% after the UAE. Similar trends have been previously observed in soybean [12,38] and chicken plasma proteins submitted to UAE [16]. Instead, in the coconut samples, the modifications of the intensities were only marginal (Figure 3C-II).

Finally, Figure 3D shows the micro-NIR spectra in the range of 900–1700 nm represented as the standard normal variate (SNV). All samples exhibited three fundamental absorption peaks at 1200–1205, 1428, and 1503 nm. The band with a maximum at 1200 (untreated samples) or 1205 nm (treated samples) may be assigned to the first harmonic stretching vibrations of the –CH groups of the protein side chains, the peak at 1428 nm to a C=C symmetric stretching, whereas the peak at 1503 nm to the first overtone of the stretching of the –OH groups [39]. The small differences observed in the UAE samples indicate only marginal modifications induced by this treatment.

### 3.8. Modifications of Techno-Functional Properties

**Figure 4:**
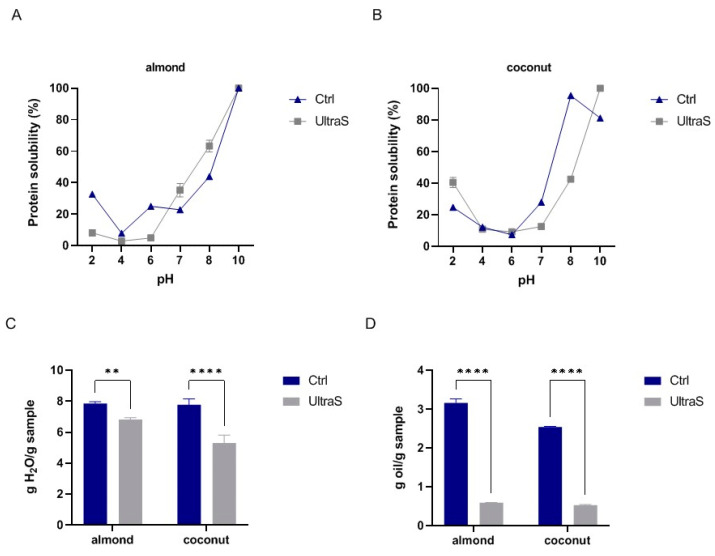
Protein solubility before and after the UAE for (**A**) almond and (**B**) coconut samples. (**C**) Water binding capacity and (**D**) oil binding capacity of untreated and treated samples. The data are represented as the means ± s.d. of three independent experiments. All data sets were analyzed by two-way ANOVA followed by Šídák’s multiple comparisons test. CTRL: control sample. (**) *p* < 0.01, (****) *p* < 0.0001.

The solubility of coconut and almond samples before and after UAE as a function of pH is shown in Figure 4A,B. The lowest solubility values of untreated (7.8%) and treated (2.9%) almond were obtained at pH 4.0, whereas in alkaline conditions, the samples were very soluble. At low pH, the UAE sample was less soluble than the untreated sample. These results agree with a recent study on wild almond [40], which showed low solubility levels around the pH 4–5 and high ones in alkaline conditions. With coconut (Figure 4B), the lowest solubility was observed at pH 6.0 either in the untreated (7.3%) or treated (9.2%) sample. Above pH 7, the solubility increased reaching the maximum value at pH 8 for the untreated sample and at pH 10 for the UAE one. These data agree with literature data [5].

The WBC and OBC are useful pieces of information for the application of these ingredients in foods. Figure 4C,D shows that in the case of almond the WBC was only slightly modified by the UAE (from 7.86 ± 0.11 g water/g sample in CtrlA to 6.89 ± 0.11 g water/g sample in UltraSA), whereas the OBC was significantly decreased (from 3.16 ± 0.10 in CtrlA to 0.59 ± 0.01 g water/g sample in UltraSA). In coconut samples, the ultrasonication either decreased the WBC or the OBC; also in this case, the most significant reduction was observed on OBC (from 2.54 ± 0.01 g water/g sample in CtrlC to 0.53 ± 0.01 g water/g sample in UltraSC (Figure 4C,D).

The solubility and water/oil binding capacity are important properties strictly correlated to other technical-functional aspects of proteins, i.e., their foaming capacity (FC) and stability (FS). Proteins in dispersion cause a lowering of the surface tension at the water/air interface, thus generating FC. The foaming properties of the proteins extracted from almond and coconut are shown in Table 3. In almond samples, which have modest foaming properties, FC and FS remained practically unchanged after the UAE. In the case of coconut, which has much better foaming properties, the treatment caused an important decrease either in the FC or in the FS (after 30 min). This reduction is probably due to extensive alterations of the physicochemical properties induced by the UAE.

### 3.9. In Vitro Protein Digestibility (IVPD)

**Figure 5:**
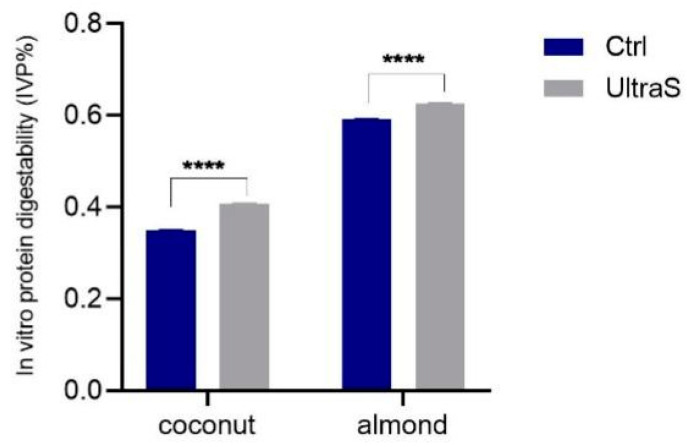
In vitro digestibility (*IVPD%*) of the same samples, (****) *p* < 0.0001.

The intense shear stress and pressure in a short time caused by the cavitation may improve the protein digestibility, owing to changes in the secondary structures as well as to disruption of the protein sequences [41]. It was thus decided to evaluate the IVPD% using a two-stage digestion with pepsin and pancreatin. The IVPD% of the raw coconut and almond samples was 35.0% and 59.2 %, respectively, whereas after UAE, the values became 40.8% and 62.6%, respectively (Figure 5). Therefore, the sonication had a favorable effect on digestibility.

The most prominent factors affecting the in vitro digestibility after processing are the protein solubility and secondary structure changes [42,43]. Digestibility is often positively affected by protein solubility, for example, an increased solubility of potato protein facilitates the interaction with digestive enzymes, leading to higher digestion rates [44]. This might happen because the ultrasound treatment exposes more hydrophilic groups by enhancing the interaction between the proteins and the water molecules. In addition, there is a negative correlation between the IVPD% and the β-sheet content [42]. Indeed, the digestibility process does not involve protein regions with a β-sheet structure and a high negative linear correlation coefficient (r = −0.980) has been found between the β-sheet contents of all proteins and the food digestibility values [45]. In our case, the increases of IVPD% induced by sonication may depend mainly on the variations in the protein secondary structures, in particular on the reduction in β-sheet substructures. 

### 3.10. Antioxidant Properties

**Figure 6:**
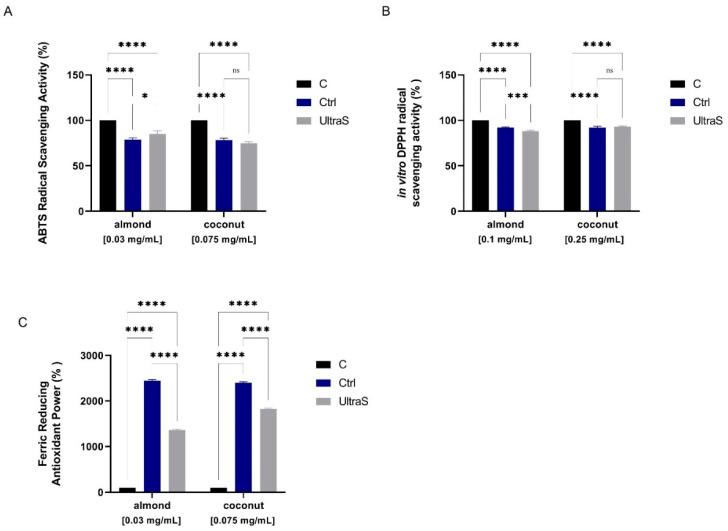
Antioxidant power evaluation of almond and coconut before and after UAE treatment: (**A**) 2,2-azino-bis-(3-ethylbenzothiazoline-6-sulfonic) acid (ABTS); (**B**), 2,2-diphenyl-1-picryl-hydrazyl (DPPH); (**C**) ferric reducing antioxidant power (FRAP) assays. The data points represent the averages ± s.d. of four independent experiments performed in duplicate. All data sets were analyzed by Two-way ANOVA followed by Šídák’s multiple comparisons test. ns: no significance. (*) *p* < 0.05; (***) *p* < 0.001; (****) *p* < 0.0001.

The antioxidant properties were assessed using different methods, such as the ABTS, DPPH, and FRAP assays. As shown in Figure 6, the CtrlA and UltraSA samples scavenged the ABTS radical by 21.24 ± 2.02% and 14.85 ± 3.34% at 0.075 mg/mL, whereas CtrlC and UltraSC samples scavenged the radical by 21.72 ± 2.18% and 25.06 ± 1.65% at 0.03 mg/mL, respectively (Figure 6A).

In the DPPH test, CtrlC and UltraSC reduced the DPPH radical by 8.06 ± 1.59% and 7.09 ± 0.94% at 0.1 mg/mL, respectively, whereas CtrlA and UltraSA by 7.99 ± 0.66% and 11.77 ± 0.96% at 0.25 mg/mL, respectively (Figure 6B). Thus, only in almond the UAE improved the ability to scavenge the DPPH radical; a similar behavior had been observed in a previous investigation on proteins extracted from soybean okara by UAE [12]. The increase may be due to the exposure of hidden amino acid residues and/or side chains with antioxidant capacities, before hidden within the three-dimensional structure of proteins.

Finally, the UAE treatment decreased the FRAP activity in a significant way either in almond samples (from 2446 ± 23.08% in CtrlA to 1362 ± 23.08% in UltraSA at 0.075 mg/mL) or in coconut samples (from 2400 ± 23.08% in CtrlC to 1823 ± 18.31% in UltraSC at 0.03 mg/mL) (Figure 6C). The reduction is probably related to the presence of chelating amino acids in the protein sequence (Cis, His, Glu) [46]. Previously (Figure 3B), we showed that the UAE reduced the content of free SH groups positioned on the surface of coconut and almond proteins. Since these groups are critical for the interaction with free radicals, their reduction in both treated samples certainly has a main role in the observed reduced FRAP ability.

## 4. Conclusions

This paper reports data on the application of UAE for improving the recovery of protein ingredients from press cakes derived from vegetal drinks manufacturing. Indeed, the protein contents of the recovered ingredients were increased by the treatment with a parallel decrement of phytic acid. The mass spectrometry analysis indicated that the UAE mainly improved the content of storage proteins. The investigation on the secondary and tertiary protein structures pointed out a decrease in β-sheet content that certainly had a role in the observed improved digestibility. As for the techno-functional properties, the WBCs of the protein ingredients were only slightly lower than those of the raw press cakes, whereas the OBCs were extremely damaged by the treatment. Different effects were observed as far as the foaming properties are involved: the modest foaming properties of almond samples were scarcely modified by the UAE treatment, whereas the good foaming properties of the coconut samples were much more sensitive. Interestingly, however, the foaming capacity was highly decreased, while the foaming stability remained practically unchanged. These materials also had moderate antioxidant properties that were only slightly modified by the UAE, with the exception of the poor results of the FRAP assay: this is probably linked to the observed reduction in free SH groups. In conclusion, in this work we have collected numerous complementary pieces of information on the protein ingredients extracted from press cakes using UAE. These results confirm that this technique is a promising strategy to be implemented at industrial level to improve the recovery and enhance the quality of protein ingredients from the by-products derived from vegetal beverage production.

## Figures and Tables

**Table 1:** Transition list of selected peptides for relative protein quantification of most abundant protein identified in coconut and almond samples.

Protein Name Accession N.	Precursor	Precursor Ion [M+2H]^2+^	Transition	Ratio (UltraS/Ctrl)
Coconut				
Legumin A0A5E4FFS0 (11S globulin)	(R)ALPDEVLQNAFR(I)	687.0	687.0 → 847.47 687.0 → 397.20	2.5
	(R)VQVVNENGDPILDDEVR(E)	637.8	637.8 → 859.45 637.8 → 540.31	6.8
	(R)NLQGQDDNRNEIVR(V)	836.1	836.1 → 630.35 836.1 → 541.27	1.9
Vicilin A0A5E4EZP4 (7S globulin)	(R)QLAFGPEMEQIFSK(Q)	812.9	812.9 → 494.29 812.9 → 743.37	1.6
	(R)EQLQALSQAASSR(R)	695.0	695.0 → 499.25 695.0 → 819.43	1.9
	(R)FEEFFPAGSR(N)	594.0	594.0 → 634.33 594.0 → 553.22	2.0
Almond				
11S globulin isoform 2 A0A0R7UCT6 (legumin)	(K)QNIGDPRR(A)	478.2	478.2 → 413.21 478.2 → 543.29	4.6
	(K)QNIGDPRRADVFNPR(G)	877.8	877.8 → 818.41 877.8 → 528.24	5.2
	(R)GGRITTLNSEK(L)	588.32	392.5 → 792.40 392. 5 → 384.23	2.6

**Table 2:** The secondary structures in almond and coconut protein assessed by CD spectroscopy.

Sample	α-Helix (%)	β-Sheet (%)	Turn (%)	Random Coils (%)
CtrlC	3.2	29.4	19.7	47.8
UltraSC	3.4 ^ns^	16.7 ^(a)^	19.1 ^ns^	60.8 ^(a)^
CtrlA	2.6	33.9	20.3	43.1
UltraSA	1.7 ^(a)^	25.2 ^(a)^	24.9 ^ns^	48.3 ^ns^

The statistical analysis was performed by T-parametric test. ^(a)^ *p* < 0.05, ^ns^: not significant versus CtrlC and CtrlA, respectively.

**Table 3:** Foaming capacity (FC) and foam stability (FS) of almond and coconut proteins.

Samples	FC (%)	FS, 5 min (%)	FS, 30 min (%)	FS, 60 min (%)
CtrlA	1.7 ± 0.8	0.5 ± 1.2	/	/
UltrSA	1.9 ± 0.6 ^ns^	1.1 ± 0.8 ^ns^	/	/
CtrlC	21.6 ± 1.9	14.1 ± 0.6	10.7 ± 1.3	3.7 ± 1.4
UltrSC	14.1 ± 0.5 ^(a)^	12.0 ± 1.8 ^ns^	6.7 ± 0.4 ^(b)^	4.6 ± 1.6 ^ns^

The statistical analysis was performed by Šídák’s multiple comparisons test. ^(a)^ *p* < 0.0001, ^(b)^ *p* < 0.01, ^ns^: not significant versus CtrlC and CtrlA, respectively.

## Data Availability

Data are contained within this article and Supplementary Materials.

## Supplementary Materials

The following supporting information can be downloaded at: https://www.mdpi.com/article/10.3390/foods11223693/s1, Table S1: Peptides identified in the samples.
